# A randomised, double-blind, sham-controlled trial of deep brain stimulation of the bed nucleus of the stria terminalis for treatment-resistant obsessive-compulsive disorder

**DOI:** 10.1038/s41398-021-01307-9

**Published:** 2021-03-29

**Authors:** Philip E. Mosley, François Windels, John Morris, Terry Coyne, Rodney Marsh, Andrea Giorni, Adith Mohan, Perminder Sachdev, Emily O’Leary, Mark Boschen, Pankaj Sah, Peter A. Silburn

**Affiliations:** 1 Systems Neuroscience Group, QIMR Berghofer Medical Research Institute, Herston, QLD Australia; 2 Neurosciences Queensland, St Andrew’s War Memorial Hospital, Spring Hill, QLD Australia; 3 Queensland Brain Institute, University of Queensland, St Lucia, QLD Australia; 4 Faculty of Medicine, University of Queensland, Herston, QLD Australia; 5 Brizbrain and Spine, the Wesley Hospital, Auchenflower, QLD Australia; 6 Centre for Healthy Brain Ageing (CHeBA), School of Psychiatry, University of New South Wales, Sydney, NSW Australia; 7 Neuropsychiatric Institute, The Prince of Wales Hospital, Randwick, NSW Australia; 8 The OCD Clinic, Bulimba, QLD Australia; 9 School of Applied Psychology, Griffith University, Gold Coast, QLD Australia; 10 Joint Center for Neuroscience and Neural Engineering, and Department of Biology, Southern University of Science and Technology, Shenzhen, Guangdong Province P. R. China

**Keywords:** Psychiatric disorders, Neuroscience

## Abstract

Deep brain stimulation (DBS) is a promising treatment for severe, treatment-resistant obsessive-compulsive disorder (OCD). Here, nine participants (four females, mean age 47.9 ± 10.7 years) were implanted with DBS electrodes bilaterally in the bed nucleus of the stria terminalis (BNST). Following a one-month postoperative recovery phase, participants entered a three-month randomised, double-blind, sham-controlled phase before a twelve-month period of open-label stimulation incorporating a course of cognitive behavioural therapy (CBT). The primary outcome measure was OCD symptoms as rated with the Yale-Brown Obsessive-Compulsive Scale (YBOCS). In the blinded phase, there was a significant benefit of active stimulation over sham (*p* = 0.025, mean difference 4.9 points). After the open phase, the mean reduction in YBOCS was 16.6 ± 1.9 points (*χ*^2^ (11) = 39.8, *p* = 3.8 × 10^−5^), with seven participants classified as responders. CBT resulted in an additive YBOCS reduction of 4.8 ± 3.9 points (*p* = 0.011). There were two serious adverse events related to the DBS device, the most severe of which was an infection during the open phase necessitating device explantation. There were no serious psychiatric adverse events related to stimulation. An analysis of the structural connectivity of each participant’s individualised stimulation field isolated right-hemispheric fibres associated with YBOCS reduction. These included subcortical tracts incorporating the amygdala, hippocampus and stria terminalis, in addition to cortical regions in the ventrolateral and ventromedial prefrontal cortex, parahippocampal, parietal and extrastriate visual cortex. In conclusion, this study provides further evidence supporting the efficacy and tolerability of DBS in the region of the BNST for individuals with otherwise treatment-refractory OCD and identifies a connectivity fingerprint associated with clinical benefit.

## Introduction

Obsessive-compulsive disorder (OCD) is a psychiatric condition with an estimated lifetime prevalence of between 1 and 2%^1^. It is characterised by the intrusion of ego-dystonic, anxiety-provoking thoughts (obsessions). These are accompanied by mental acts or behaviours (compulsions), which must be carried out to neutralise the obsessions, or to mitigate anxiety associated with them^2^. Remission of symptoms with pharmacological treatment is rare^3^ and persistent impairment is relatively common even with combination therapy^4^. Psychological treatment is often intolerable for those with a severe illness: deliberate exposure to obsessive thoughts during cognitive behavioural therapy (CBT) is aversive and distressing^5^. These factors mean that OCD is a chronic disorder with a detrimental effect on functioning across the lifespan, making it a leading neuropsychiatric cause of global disability^6^.

Deep brain stimulation (DBS) is a reversible and adjustable form of targeted neuromodulation that has been used successfully for the treatment of movement disorders such as Parkinson’s disease for over 25 years^7,8^. DBS was first employed for the treatment of intractable OCD in the late 1990s^9^, with initial surgical targeting in the anterior limb of the internal capsule (ALIC) informed by prior work using ablative neurosurgery^10^. Further work reproduced these encouraging preliminary outcomes^11–14^, finding improved response with posterior migration of the target within the ventral capsule and ventral striatum^15^. The anteromedial segment of the subthalamic nucleus (STN) has also been a promising target for neuromodulation^16^. More recently, two randomised, placebo-controlled, crossover trials of DBS at the nucleus accumbens (NAcc)/ALIC interface^17^ and the BNST/ALIC interface^18^ demonstrated a statistically-significant benefit of active stimulation over sham. Subsequently, the efficacy of NAcc/ALIC DBS has been further supported in larger open-label studies^19,20^.

The clinical benefits (and side effects) of DBS for movement disorders arise not only from the effect of focal stimulation at the target nucleus, but also from the modulation of distributed brain networks structurally and functionally connected to the stimulation field^21–26^. In a similar manner, brain networks associated with response to DBS for OCD can be delineated. In prior work, reduction in OCD symptoms 12-months after NAcc/ALIC DBS was associated with connectivity of the stimulation site with the right ventrolateral prefrontal cortex, with a fibre tract predictive of symptom reduction identified in the ventral ALIC bordering the BNST^27^. A randomised trial directly comparing ALIC and anteromedial STN stimulation found both to be clinically effective targets but with distinct structural connectivity profiles and dissociable effects on mood and cognitive flexibility^28^. However, a pooled analysis of four cohorts employing either STN or ALIC stimulation identified a universal tract associated with the clinical response that could predict outcome in an out-of-sample cross-validation^29^. This tract traversed both the anteromedial STN and ventral ALIC, projecting to ventrolateral prefrontal cortex. Overall, these findings suggest that different surgical targets may act to reduce OCD symptoms through modulation of a shared network, whilst change amongst more fine-grained behavioural endophenotypes may result from modulation of networks that are not shared between targets^30^.

In this study, using a randomised, double-blind, sham-controlled, staggered-onset design, we investigate the effects of DBS at the BNST/NAcc interface in a sample of Australian participants with severe, treatment-resistant OCD. We delineate the structural connectivity profile of effective stimulation and compare this with the aforementioned prior work. Therefore, we provide both placebo-controlled data (which is considered to reduce bias and improve the reliability of experimental findings) and data on circuit-specific neuromodulation associated with clinical improvement. We also add CBT incorporating exposure and response prevention (ERP) to the open phase of the trial, in order to investigate whether this is now tolerable for our participants and leads to an additive clinical response, as has been identified in a previous cohort^31^. We report outcomes during the blinded phase and after one year of open stimulation following completion of CBT.

## Materials and methods

### Participants

All procedures were carried out in accordance with the experimental protocol approved by the Human Research Ethics Committees of the University of Queensland and UnitingCare Health. Participants aged 18–70 with severe, treatment-resistant OCD of at least five years duration were referred by their treating psychiatrists and evaluated independently by two psychiatrists in the research team (PEM and RM). The diagnosis of OCD was confirmed according to criteria defined by the Diagnostic and Statistical Manual of Mental Disorders, fifth edition (DSM-V)^2^. Severity was denoted by a mean score of at least 24 on the Yale-Brown Obsessive-Compulsive Scale (YBOCS)^32^, measured twice at least 2 weeks apart by separate investigators. Treatment refractoriness was defined by insufficient response to at least: (i) two trials of selective serotonin reuptake inhibitors at maximum tolerated dose for at least 12 weeks, (ii) one trial of clomipramine at maximum tolerated dosage for at least 12 weeks, plus (iii) one augmentation trial with an antipsychotic for at least eight weeks in combination with one of the aforementioned drugs, plus iv) one complete trial of ERP-based CBT confirmed by a psychotherapist. Exclusion criteria included pregnancy, a past history of a chronic psychotic or bipolar disorder, severe personality disorder, suicidality in the previous 12 months, substance use disorder (except tobacco), major neurological comorbidity or severe head injury, prior ablative neurosurgery and current implanted cardiac pacemaker, defibrillator or other neurostimulator. Suitable and consenting candidates were approved by an independent Mental Health Review Tribunal prior to neurosurgery. Prior to implantation of the first participant, the trial was registered on the Australian and New Zealand Clinical Trials Registry (Universal Trial Number: U1111-1146-0992).

### Device implantation

Bilateral implantation of Medtronic 3389 quadripolar electrodes took place in a single-stage procedure using a CRW stereotactic apparatus based on preoperative structural magnetic resonance neuroimaging (Supplementary Materials). The most ventral contact was sited posterior and inferior to the NAcc in the region of the lateral hypothalamus, with contacts selected to distribute stimulation within the BNST approaching the posterior border of the NAcc. Targeting was performed manually by P.A.S. from the T1 weighted imaging, using the caudate, nucleus accumbens and optic tract as anatomical landmarks. Postoperative lead placement was confirmed with CT imaging. Electrodes were connected to an Activa PC + S implantable pulse generator (IPG) in either the pectoral or abdominal fascia. Analysis of long-term, ambulatory electrophysiological data will be reported in forthcoming work.

### Timeline, assessment and intervention

Following device implantation, participants entered a one-month recovery phase during which all stimulators were off. Thereafter, participants began a 3-month period during which their stimulators were either turned on or remained switched off whilst both participants and assessors were blinded to status. After this, participants continued in an open-label (unblinded) trial where all stimulators were on. Assessments took place at baseline one week before surgery, fortnightly in the recovery phase, monthly in the blinded phase and monthly for the first three months of the open phase, with the time between assessments subsequently extending to two and then 3 months. The primary outcome measure was OCD severity as assessed by the YBOCS score, derived from a ten-item semi-structured interview assessing obsessions and compulsions, with a maximum score of 40. Depressive symptoms were assessed as a secondary outcome with the Montgomery Åsberg Depression Rating Scale (MADRS) score, derived from a ten-item semi-structured interview with a maximum score of 60^33,34^. Participants were referred for a ten-session course of ERP-based CBT with a clinical psychologist (EOL or MB) during the open phase once DBS parameters had been optimised and YBOCS reduction had plateaued.

Stimulation was commenced in an identical manner for participants regardless of whether they were turned on in either the blinded or open-label phase (participants turned on in the blinded phase continued active stimulation at the same settings in the open phase). Contact 1 (left hemisphere) and contact 9 (right hemisphere) were selected with an initial stimulation amplitude of 1 Volt, a pulse-width of 90 microseconds and a frequency of 130 Hertz. Stimulation was increased at weekly to fortnightly intervals in increments of 0.5–1 Volt to a target of 4.5 Volts. The target amplitude was selected based on amplitudes employed in prior work. Stimulation settings were symmetric between hemispheres. If there was a relative lack of response as assessed with the YBOCS, additional stimulation changes were trialled: including further increases in amplitude in 0.1 Volt increments, a trial of a pulse-width of 120 microseconds or the activation of a second contact on each electrode. Psychotropic medications were unchanged throughout the trial unless requested for clinical reasons by the participant’s usual psychiatrist.

### Randomisation and blinding

Participants were randomly allocated in a 1:1 ratio to ‘on’ or ‘off’ groups in the blinded phase by an external statistician, using an online tool (https://www.sealedenvelope.com). Only the lead neurologist (PAS) and programming psychiatrist (PEM) were informed of the allocation. The psychiatrist assessing primary and secondary outcomes (RM) remained blinded to participant status. To reduce the likelihood of participants becoming unblinded by sensations associated with active stimulation, no contact testing was performed and the slow titration protocol was followed in all cases.

### Statistical analysis

Data analysis was performed in the R software environment^35^. In the blinded phase of the trial, the mean change in YBOCS and MADRS score was compared between groups with a two-sample *t*-test. After one year of open stimulation and following a course of CBT, the reduction in YBOCS and MADRS score was assessed with the package *lmerTest*^36^ using a random-intercept, random-slope, linear mixed-effects model incorporating demographic variables and baseline severity:

$${\mathrm{YBOCS}}\,{\mathrm{Score}}_{ij}\sim {\mathrm{TimeSinceDBS}}_{ij} + {\mathrm{Age}}_i + {\mathrm{Gender}}_i + {\mathrm{YBOCS}}\,{\mathrm{Baseline}}_{ij} + \left( {1|{\mathrm{ID}}} \right) + \left( {1|{\mathrm{TimeSinceDBS}}_{ij}} \right)$$

with _*i*_ denoting participant and _*j*_ denoting timepoint and the term in bold (the accrued effect of DBS over time on obsessive and depressive symptoms) being the coefficient of interest. Hypothesis testing on a null model (omitting TimeSinceDBS) was performed with the *anova* function in the *lavaan* package.

Consistent with prior work, participants were defined as responders for OCD and depression if they attained a reduction of 35% in YBOCS score and 50% in MADRS score, respectively.

### Electrode localisation and volume of tissue activation

Neuroimaging acquisition parameters are supplied in Supplementary Material. DBS electrodes were localized using the Lead-DBS toolbox version 2.2 (https://github.com/netstim/leaddbs/tree/develop)^37,38^. Preoperative structural acquisitions were co-registered with postoperative CT imaging and then normalized into common ICBM 2009b nonlinear asymmetric space using the SyN approach implemented in advanced normalization tools (ANTs)^39^. Electrode trajectories were reconstructed after correcting for brainshift in postoperative acquisitions by applying a refined affine transform in a subcortical area of interest calculated pre- and postoperatively. For each electrode, a volume of activated tissue (VAT) was estimated using a volume conductor model of the DBS electrode and surrounding tissue, based on each participant’s individualised stimulation settings and a finite element method to derive the gradient of the potential distribution^38^. An electric field (E-field) distribution was also modelled^40^.

### Structural connectivity estimation and YBOCS reduction

Three methods were used to assess the relationship between the structural connectivity of the stimulation field and the primary outcome measure. Firstly, using the Lead-DBS toolbox, each participant’s VAT in each hemisphere was integrated with a normative whole-brain structural connectome incorporating six million fibres derived from 985 participants in the Human Connectome Project who had undertaken multi-shell diffusion-weighted imaging^41^. Fibres traversing each participant’s VAT were selected from the group connectome based on the E-field gradient strength (i.e. fibres in peripheral VAT regions with a low E-field were down-weighted) and projected to the volumetric surface of the ICBM 2009b nonlinear asymmetric brain in 1 mm isotropic resolution. A connectivity profile for each participant was expressed as the weighted number of fibre tracts between the stimulation site and each brain voxel. Subsequently, each voxel on the corresponding connectivity profile was correlated with clinical improvement on the YBOCS score using a Spearman rank correlation coefficient, forming an ‘R-map’. Combined across all participants, these maps identify regions to which strong connectivity is associated with good clinical outcome, modelling ‘optimal’ connectivity from the stimulation field to the rest of the brain^21^. To verify these findings, the data were cross-validated in a leave-one-out design. Each participant was sequentially excluded and the optimal connectivity profile was computed on the remaining participants. Subsequently, YBOCS reduction was predicted for the excluded participant based on comparison between individual and group connectivity estimates (using a Fisher z-transformed spatial correlation coefficient) and the empirical outcome was correlated with the predicted outcome derived from the remaining sample.

Secondly, individual fibres associated with YBOCS reduction were identified. Each whole-brain fibre was tested across the cohort between participants with a stimulation volume that encompassed the fibre (connected) and those where the fibre did not traverse the volume (unconnected). If there was a significant difference between YBOCS reduction in participants with connected and unconnected VATs (using a two-sided, two-sample *t*-test), then this fibre was identified as discriminative of outcome. This process yielded a ‘fibre *t*-score’, with high-values indicating that this fibre was strongly discriminative of clinical outcome^27^. Only the top 5% of fibres positively correlated with the primary outcome variable were selected for analysis to mitigate the risk of false positive associations.

Finally, to explore whether connectivity to specific cortical regions was related to YBOCS reduction, a region of interest analysis was informed by findings from the aforementioned methods. Cortical parcellations were derived from the Desikan-Killiany-Tourville labelling protocol^42,43^, with connectivity estimates between each VAT and cortical region entered into the multivariate linear mixed-effects model to derive an estimate of effect size and statistical significance.

### Replication of prior connectomic data

Following a recent replication study^44^, we tested whether clinical response to DBS in our cohort was associated with the recruitment of white matter fibres in a unified connectomic tract developed from a pooled analysis of response to STN or ALIC stimulation for OCD^29^. In this analysis, fibres from the atlas traversing the VAT of each participant were weighted by the e-field as described above and the extent of tract recruitment was used to predict empirical improvement. This analysis was facilitated by the authors of the original study, who shared their data.

## Results

### Participants

**Fig. 1:**
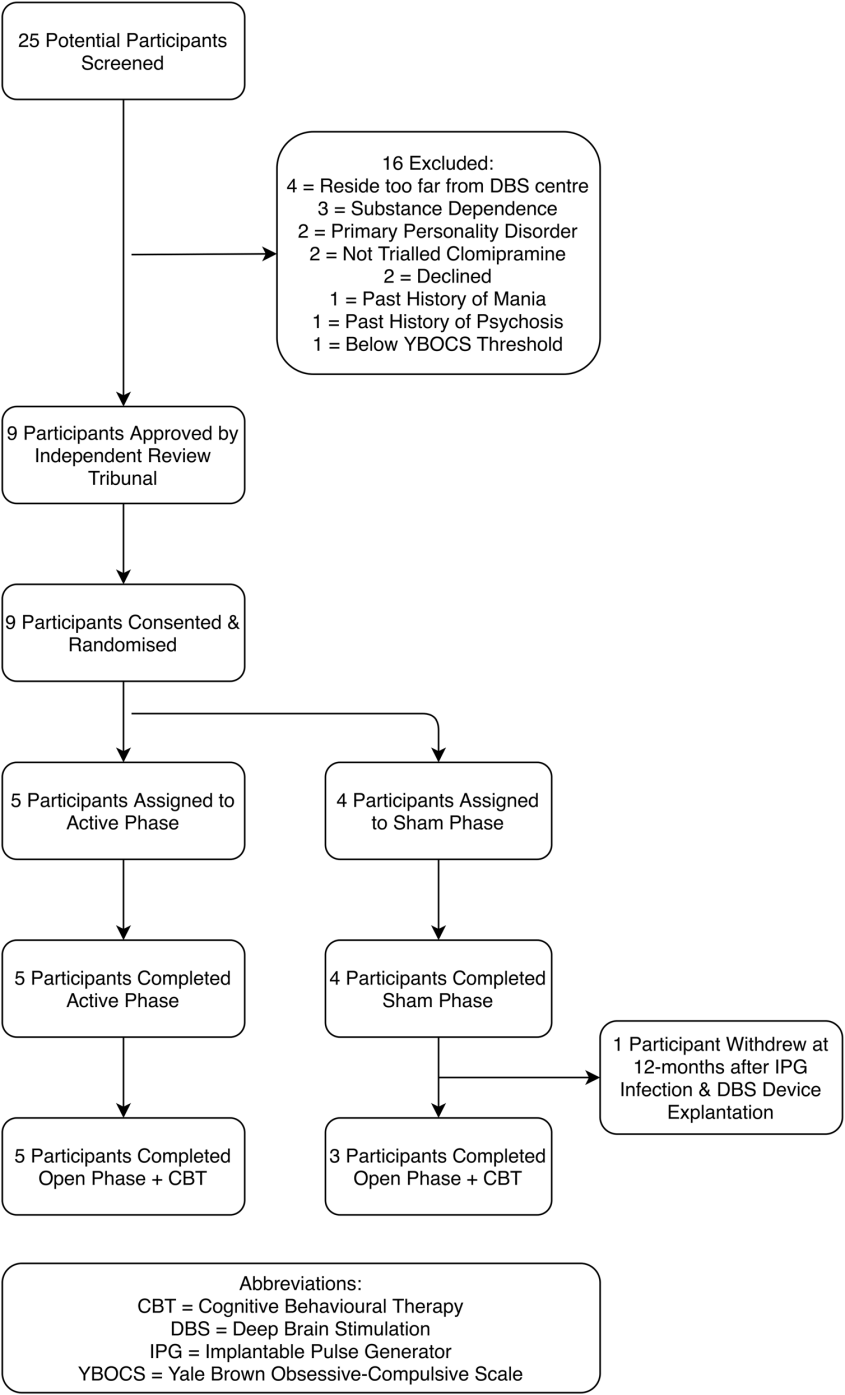
Flow diagram of participant recruitment, randomisation and treatment.

**Fig. 2:**
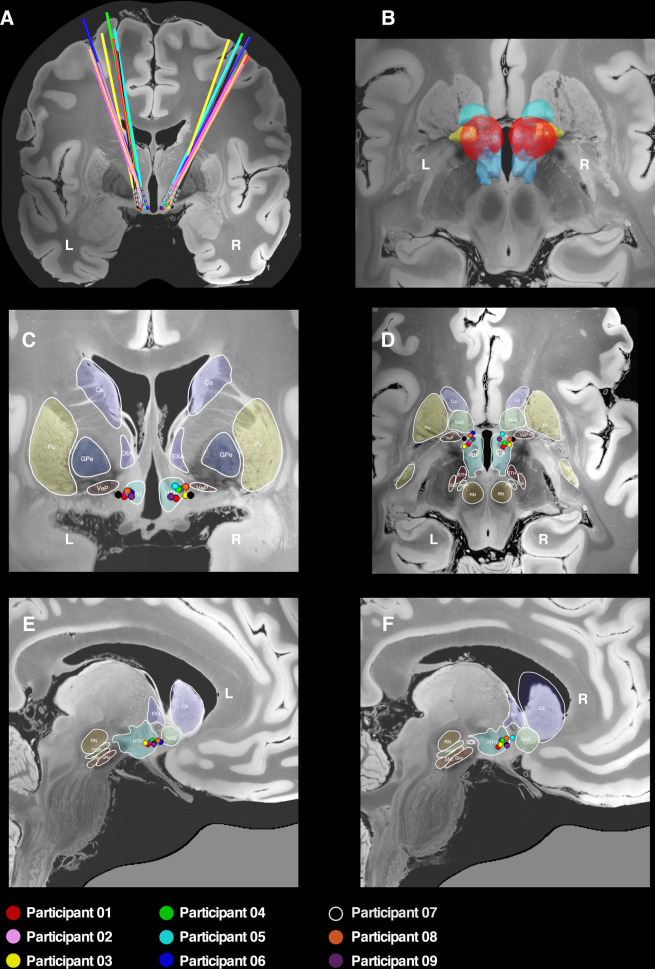
Localisation of electrodes and active contacts. DBS electrodes were localised with the Lead-DBS toolbox and represented in common ICBM 2009b nonlinear asymmetric space incorporating a 7-Tesla MRI at 100 micron resolution^73^ with subcortical parcellations derived from a recent high-resolution atlas^74^. **A** Three-dimensional reconstruction in the coronal plane showing electrode trajectories for the nine participants. **B** Three-dimensional reconstruction in the axial plane showing the distribution of the aggregated stimulation field across the cohort (red), which can be seen to encompass the posterior segment of the nucleus accumbens (light green), the ventral pallidum (yellow) and the hypothalamus (blue). **C** Two-dimensional reconstruction of active contacts in coronal plane. **D** Two-dimensional reconstruction of active contacts in axial plane. **E**, **F** Two-dimensional reconstruction of active contacts in sagittal plane. In the two-dimensional representations, coloured circles represent the second most inferior contact on each electrode (i.e. contact 9 on right electrode and contact 1 on left electrode). Ca caudate, EXA extended amygdala (BNST), GPe globus pallidus external segment, HTH hypothalamus, NAC nucleus accumbens, PBP parabrachial pigmented nucleus, Pu putamen, SN substantia nigra, STH subthalamic nucleus, RN red nucleus, VeP ventral pallidum.

Nine participants (all right-handed, four females, mean age 47.9 ± 10.7 years, mean baseline YBOCS 32.7 ± 2.6) were recruited, randomised and implanted (Fig. 1 and Table 1). Contacts selected for activation were located in the superolateral region of the hypothalamus with electrical stimulation distributed in the BNST posterior to the NAcc and inferomedial to the ventral pallidum (Fig. 2 and representative planning/postoperative imaging for one participant in Supplementary Fig. 1). One participant (05) developed an acute implantation effect with a reduction in the intensity of obsessive thoughts for 72 h postoperatively, before returning to baseline. All participants completed the blinded phase. Five participants were randomised to active treatment and four to sham. Only one participant (02) randomised to active treatment did not reach the target amplitude of 4.5 Volts during the blinded phase due to mild agitation at higher amplitudes (an internal sense of anxiety described as akin to consuming excess caffeine, but with no observable behavioural dysregulation). Given the robust symptom reduction observed in this participant, a lower amplitude was selected for chronic stimulation. One participant (05) appeared to show a placebo response to sham stimulation with a 20 percent reduction in YBOCS score during the blinded phase (Supplementary Table 1). During the open phase, one participant (05) developed an IPG infection necessitating DBS device explantation and exit from the trial. Scores at trial exit were carried forward for the two remaining data points. The eight remaining participants completed a course of ERP-based CBT. One participant (06) switched antidepressants and antipsychotics during the trial due to non-response to DBS and persistence of clinically-significant symptoms. Stimulation parameters were modified during the open phase to optimise response in some participants (Table 1). In five participants (01, 04, 07, 08 and 09) a second contact was activated on each stimulating electrode. In three participants (04, 06 and 09) the stimulation amplitude was increased above 4.5 Volts. In one participant (06) the pulsewidth was increased.

**Table 1:** Details of participants.

Participant age and gender	OCD phenomenology; age of onset; previous therapies^a^ ; comorbidities and YBOCS at baseline	Psychotropic medication and chronic stimulation parameters^b^	Percentage YBOCS and MADRS reduction at end of open phase	Serious adverse events	Adverse events
1 28 Female	Contamination Onset age 10 4 antidepressants / 3 antipsychotics Major depressive disorder YBOCS 32	Clomipramine 150 mg Quetiapine 100 mg Olanzapine 5 mg DBS Right hemisphere: C+ 8–9–4.5 V/90 µs/130 Hz DBS Left hemisphere: C+ 0–1–4.5 V/90 µs/130 Hz	YBOCS = 53.1 MADRS = 57.1	Nil	Parasomnia (sleepwalking)
2 29 Male	Harming others/Sexuality/Blasphemy Onset age 9 16 antidepressants / 3 antipsychotics/ECT Major depressive disorder YBOCS 33	Tranylcypromine 30 mg Nortriptyline 75 mg Diazepam 5 mg DBS Right hemisphere: C+ 9–3.5 V/90 µs/130 Hz DBS Left hemisphere: C+ 1–3.5 V/90 µs/130 Hz	YBOCS = 69.7 MADRS = 70.6	Deviation of one DBS electrode during implantation requiring removal and reimplantation.	Pustule at IPG site Lead tightening behind ear Reduced libido Mild agitation at higher stimulation amplitudes
3 57 Male	Sexuality Onset age 19 12 antidepressants / 7 antipsychotics Nil YBOCS 29	Clomipramine 50 mg DBS Right hemisphere: C+ 9–4.5 V/90 µs/130 Hz DBS Left hemisphere: C+ 1–4.5 V/90 µs/130 Hz	YBOCS = 51.7 MADRS = 50.0	Nil	Nil
4 54 Male	Harming others Onset age 16 9 antidepressants / 4 antipsychotics / ECT Major depressive disorder YBOCS 35	Clomipramine 200 mg Quetiapine XR 400 mg Clonazepam 1.5 mg DBS Right hemisphere: C+ 9–10–4.7 V/90 µs/130 Hz DBS Left hemisphere: C+ 1–2–4.7 V/90 µs/130 Hz	YBOCS = 54.3 MADRS = 35.3	Two inpatient psychiatric admissions to manage recurrence of depressive symptoms	Nil
5 57 Female	Sexuality/Symmetry Onset age 6 9 antidepressants / 4 antipsychotics / ECT / rTMS Major depressive disorder / body dysmorphic disorder YBOCS 32	Sertraline 100 mg Pregabalin 150 mg Clonazepam 0.25 mg DBS Right hemisphere: C+ 9–4.5 V/90 µs/130 Hz DBS Left hemisphere: C+ 1–4.5 V/90 µs/130 Hz	YBOCS = 28.1 MADRS = 46.2	Infection of IPG requiring DBS device explantation	Nil
6 47 Female	Contamination Onset aged 8 19 antidepressants / 5 antipsychotics / ECT / rTMS Major depressive disorder YBOCS 28	Tranylcypromine 10 mg Imipramine 50 mg Clonazepam 0.5 mg Olanzapine 10 mg Quetiapine IR 100 mg Lithium XR 450 mg DBS Right hemisphere: C+ 10–5.6 V/120 µs/130 Hz DBS Left hemisphere: C+ 1–5.6 V/120 µs/130 Hz	YBOCS = 0 MADRS = −4.0	Five inpatient psychiatric admissions to manage persistence of obsessive & depressive symptoms	Nil
7 54 Male	Doubt/Perfectionism Onset aged 7 3 antidepressants / 2 antipsychotics Nil YBOCS 35	Clomipramine 50 mg Sertraline 250 mg DBS Right hemisphere: C+ 9–10–4.5 V/90 µs/130 Hz DBS Left hemisphere: C+ 1–2–4.5 V/90 µs/130 Hz	YBOCS = 48.6 MADRS = 80.0	Nil	Nil
8 48 Female	Checking/Magical thinking Onset aged 5 8 antidepressants / 3 antipsychotics/ECT Major depressive disorder YBOCS 34	Clomipramine 125 mg Desvenlafaxine 200 mg Olanzapine 5 mg DBS Right Hemisphere: C+ 9–10–4.5 V/90 µs/130 Hz DBS Left hemisphere: C+ 1–2–4.5 V/90 µs/130 Hz	YBOCS = 82.3 MADRS = 78.9	Nil	Nil
9 55 Male	Checking/Doubt Onset aged 7 5 antidepressants / 2 antipsychotics Nil YBOCS 36	Fluoxetine 80 mg Dexamphetamine 60 mg DBS Right hemisphere: C+ 9–10–5.0 V/90 µs/130 Hz DBS Left hemisphere: C+ 1–2–5.0 V/90 µs/130 Hz	YBOCS = 58.3 MADRS = 77.8	Nil	Nil

*ECT* electroconvulsive therapy, *IPG* implantable pulse generator, *IR* immediate release, *MADRS* Montgomery Åsberg Depression Rating Scale, *rTMS* repetitive Transcranial Magnetic Stimulation, *XR* extended release, *YBOCS* Yale-Brown Obsessive-Compulsive Scale.

^a^For brevity, details of past psychotherapies not listed here.

^b^On the quadripolar electrode, contacts are numbered 8–11 in the right hemisphere and 0–3 in the left hemisphere.

### Outcomes

**Fig. 3:**
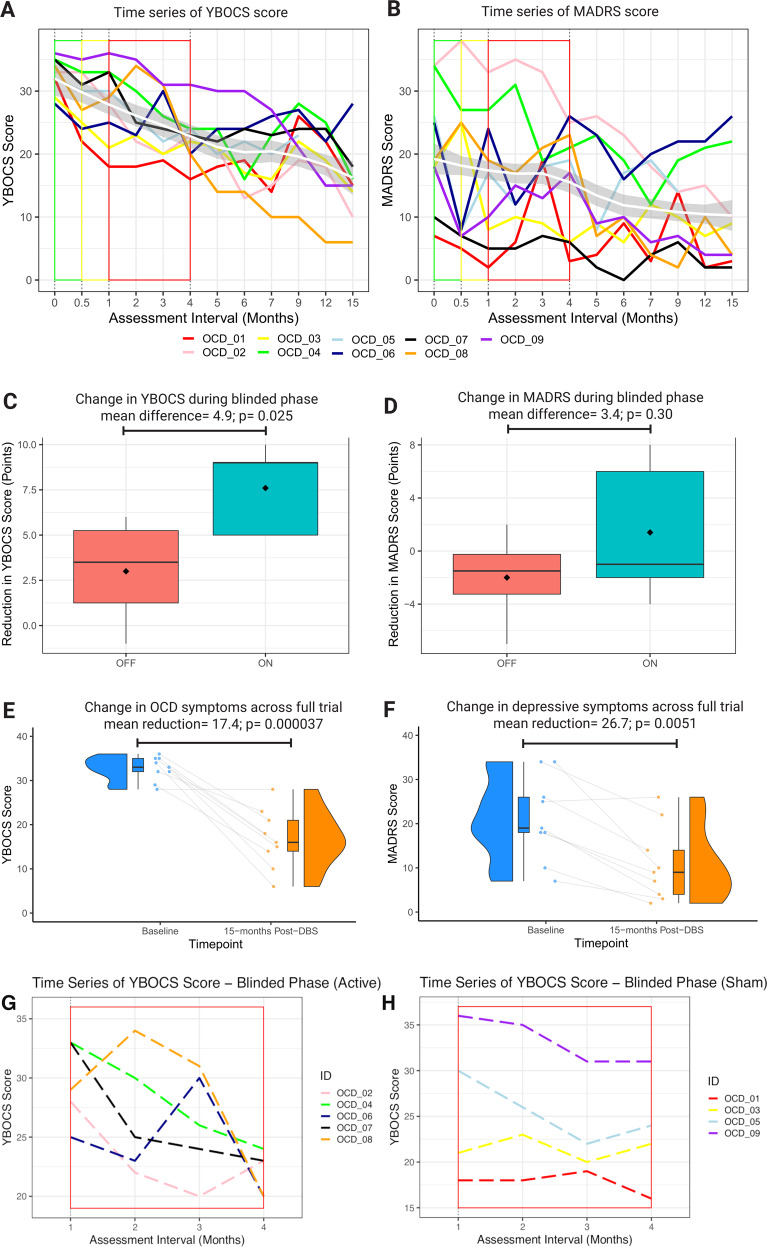
Participant Outcomes. **A**, **B** Time series of individual participant outcomes for primary (YBOCS) and secondary (MADRS) variables. Within each graph, group-average trajectory is represented by a loess smoothed curve (white) ± 1 standard error (grey). Baseline measurement denoted by green outline, recovery phase by yellow outline and blinded phase by red outline. **C**, **D** Boxplots of YBOCS and MADRS change by randomised group (on = green versus off = red) during the blinded phase. **E**, **F** Raincloud plots of YBOCS and MADRS change across the full trial. Raincloud plots made with code provided by Allen et al.^75^. and van Langen^76^. **G**, **H** Trajectories of the participants by group (active versus sham) in the blinded phase. There were five participants in the active group and four in the sham group.

A Shapiro–Wilk test indicated a normal distribution of the data in both blinded (W = 0.93, *p* = 0.44) and open (W = 0.97, *p* = 0.91) phases. In the blinded (on versus sham) phase, there was a statistically-significant difference in YBOCS reduction in favour of active stimulation (*t* = −2.9, *p* = 0.025, mean difference 4.9 points, 95 % CI = 0.8–8.9) (Fig. 3 including individual outcome data and Supplementary Table 1). There was no significant difference in MADRS reduction (*t* = −1.1, *p* = 0.30, mean difference 3.4 points, 95 % CI = −3.7–10.5).

After 1 year of open-label stimulation and a course of ERP-based CBT, the mean reduction in YBOCS was 17.4 ± 2.0 points (*χ*^2^ (11) = 39.9, *p* = 3.7 × 10^−5^) with no statistically-significant covariates (Fig. 3). Seven participants were responders as defined by the 35% YBOCS reduction criterion, with a mean percentage reduction across the cohort of 49.6 ± 23.7. ERP-based CBT commenced an average of 10.1 ± 2.6 months after DBS with a mean additive YBOCS reduction of 4.8 ± 3.9 points (*t* = −3.5, *p* = 0.011, 95% CI = 1.5–8.0). The mean reduction in MADRS was 10.8 ± 2.5 points (*χ*^2^ (11) = 26.7, *p* = 0.0051) with age (*t* = −2.7, *p* = 0.0084) and baseline MADRS (*t* = 13.4, *p* = 2.0 × 10^−16^) being significant covariates. Six participants were responders as defined by the 50% MADRS reduction criterion, with a mean percentage reduction across the cohort of 54.7 ± 27.2.

### Relationship of structural connectivity to YBOCS reduction

**Fig. 4:**
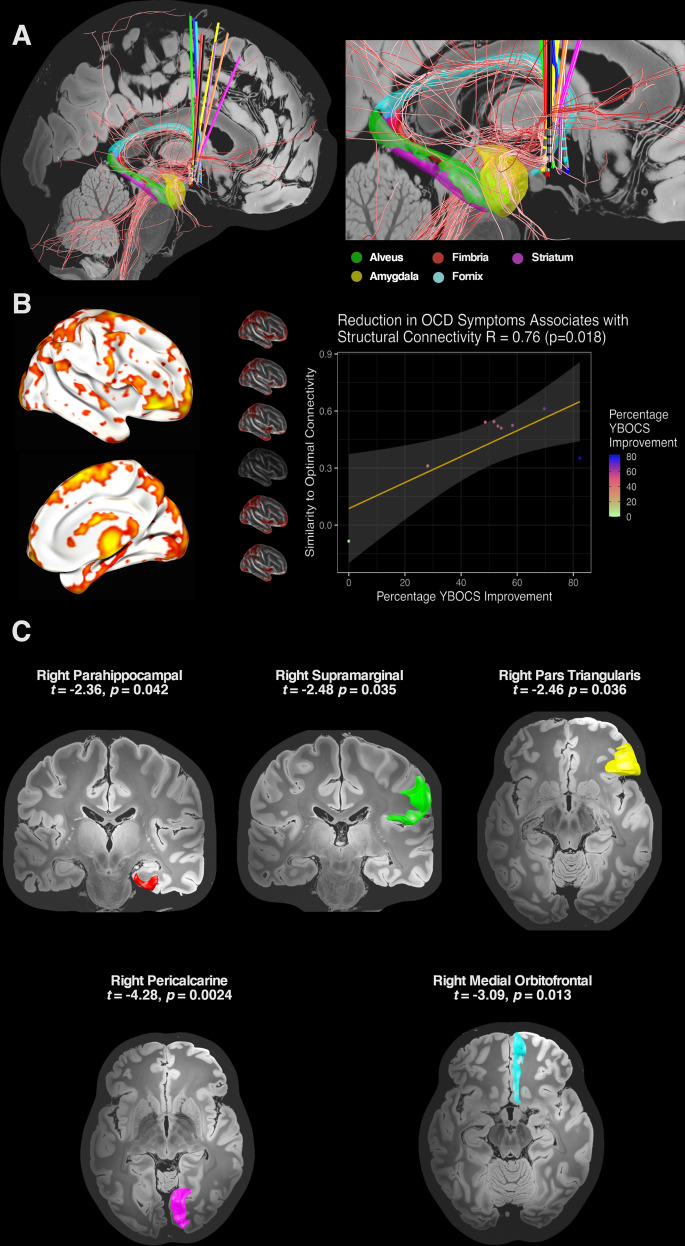
Structural connectivity and YBOCS reduction. **A** White matter fibres connected to the stimulation field and discriminative of outcome were isolated in the right hemisphere. These included a fibre tract passing through the midbrain to the ventrolateral prefrontal cortex and a fibre tract connecting the site of stimulation with the amygdala. Fibres in this region also passed through the hippocampal white matter and returned to the BNST via the stria terminalis adjacent to the fornix. Subcortical parcellations of the amygdala, hippocampus and fornix were derived from recent automated segmentation methods^77–79^. Additional views presented in Supplementary Fig. 4. **B** An optimal connectivity profile was generated by identifying those brain voxels structurally connected with the stimulation field and most highly correlated with YBOCS reduction. Cortical regions implicated in this optimal right-hemispheric ‘R-map’ included ventromedial and ventrolateral prefrontal cortex, dorsomedial prefrontal cortex, medial temporal cortex, parietal cortex and extrastriate visual cortex. These findings were corroborated in a leave-one-out cross-validation, in which each participant’s percentage YBOCS reduction was predicted by comparing their structural connectivity profile with an optimal connectivity map derived from the remaining participants. **C** In a region of interest analysis, cortical regions derived from the R-map were tested in a multivariate linear mixed-effects model for their association with YBOCS reduction.

The local dispersion of the stimulation field within neighbouring subcortical structures, including the NAcc, ventral pallidum, hypothalamus and terminal fibres of the stria terminalis was not related to relief of OCD symptoms (plotted in Supplementary Figs. 2 and 3). Using a normative connectome to identify white matter fibres connected to the stimulation field in each hemisphere for each participant, those connections most highly associated with YBOCS reduction were found in the right hemisphere (Fig. 4A and additional views Supplementary Fig. 4). These included a tract passing through the midbrain, traversing the BNST and onwards to the right ventrolateral prefrontal cortex. A tract connecting the BNST with the right amygdala was also identified, with connecting fibres passing through the hippocampal white matter and traversing back into the BNST via the fornix.

An ‘optimal’ connectivity map derived from correlating each brain voxel (weighted by structural connectivity) to YBOCS reduction also identified the right ventrolateral and parahippocampal regions, as well as right extrastriate, parietal and dorsomedial prefrontal areas (Fig. 4B and local maxima Supplementary Table 2). In a leave-one-out cross-validation, structural connectivity of the stimulation field was significantly associated with YBOCS reduction (*r* = 0.76, *p* = 0.018).

Based on these findings, corresponding cortical regions derived from the Desikan-Killiany-Tourville labelling protocol were entered into the multivariate, linear mixed-effects model. Structural connectivity of the right-hemispheric stimulation field with right orbitofrontal (*t* = −3.1, *p* = 0.013), right parahippocampal (*t* = −2.4, *p* = 0.042), right pars triangularis (*t* = −2.5, *p* = 0.036), right pericalcarine (*t* = −4.3, *p* = 0.0024) and right supramarginal regions (*t* = −2.5, *p* = 0.035) was significantly associated with YBOCS reduction. Connectivity with the right paracentral (*t* = −1.8, *p* = 0.11) region was not statistically-significant. Univariate correlations displayed in Supplementary Material.

Finally, the association of clinical response with recruitment of a white matter tract previously identified from a large pooled analysis of ALIC and STN DBS for OCD^29^ was tested. A non-significant trend for the recruitment of right-hemispheric fibres was identified (*r* = 0.47, *p* = 0.21, plotted in Supplementary Fig. 5).

### Adverse events

There were nine serious adverse events (SAEs) affecting four participants (Table 1). Five of these were attributable to one participant (06) who was a non-responder and was readmitted to hospital to manage persistent psychiatric symptoms. A further participant (04) was readmitted to hospital on two occasions to manage a recurrence of depressive symptoms. Two SAEs were device related. One participant (02) required re-siting of a DBS electrode that had migrated 3 mm from the target during implantation. This was accomplished without any further complication. One participant (05) developed an infection of the IPG that migrated to the extension leads necessitating removal of the DBS device. Unfortunately, this participant experienced a return of OCD symptoms to baseline levels and remains in contact with the clinical team, being desirous of future device reimplantation when feasible. There were five adverse events affecting two participants (Table 1). These were transient in nature except for reduced libido (participant 02), which persisted throughout follow up. Notably, there were no serious psychiatric adverse effects considered to be device related. All participants (except 05 who withdrew) required IPG replacement due to battery depletion during the study (although a non-responder, at the time of battery depletion participant 06 requested IPG replacement to allow therapy to continue in the hope that a response would eventuate given further time).

## Discussion

In nine participants with severe, treatment-refractory OCD, we demonstrate that DBS of the BNST region substantially alleviated symptoms, with a mean YBOCS reduction of 49.6% and seven participants meeting the threshold for clinically-significant response after 12-months of open-label stimulation. Moreover, we describe a statistically-significant benefit of active stimulation over sham during a 3-month, double-blind, delayed-onset phase. Our data add to the emerging literature supporting the use of DBS as a therapy in otherwise treatment-resistant OCD and specifically reproduces prior work targeting the BNST^18^.

It must be noted that the effect size in the blinded phase is considerably smaller than in open-label treatment (4.9 points versus 17.4 points). There are several factors that may account for this. First, the blinded phase was considerably shorter than the open phase and occurred at the commencement of follow up, before stimulation settings had been optimised (pros and cons of this approach discussed further below). In particular, due to the slow titration protocol, participants spent only the latter stages of this blinded phase at higher stimulation amplitudes. Second, the on stimulation group in the blinded phase included participant 06, a non-responder, which reduced the effect size. Third, the open phase incorporated ERP-based CBT, which we and others believe is an important additional step in challenging fear conditioning (discussed below). Other sham-controlled trials of DBS in OCD have reported larger effects in the blinded phase (12 points in Luyten et al.^18^, 9 points in Mallet et al.^16^, and 8 points in Denys et al.^17^) but importantly all of these studies have used a crossover design after an open-label phase of stimulation optimisation. Whilst there are many benefits to this approach, one advantage of our design is that participants were not exposed to stimulation prior to the blinded phase, thereby reducing the likelihood that a participant could recognise sensations associated with active stimulation and vice versa. Our mean YBOCS reduction in the open-label phase at 12-months (49.6%) was equivalent to recent open-label studies targeting neighbouring brain structures in the ALIC (Denys et al. 40%^19^ and Menchón et al. 42%^20^).

Open-label stimulation also significantly reduced co-morbid depressive symptoms, although this result should be viewed with more circumspection as depression was not a primary target of the intervention and two participants reported only mild symptoms at baseline. This heterogeneity in depression severity at baseline reduced the power to detect a significant difference during the sham-controlled phase, although depressive symptoms in the cohort were significantly reduced during the longer open-label phase. It could be hypothesised that mood improved here as a secondary phenomenon subsequent to reduction in OCD severity and an improvement in functioning, although further data in a larger cohort (including the collection of data related to global functioning such as a GAF score) will be necessary to address this question.

Nine participants is a small sample size for a clinical trial but is consistent with other clinical trials of DBS for treatment-resistant psychiatric indications. It must be emphasised that to be eligible for these studies, participants must typically have a lifelong, severe and intractable illness that has been unresponsive to other evidence-based therapies. Therefore, the number of eligible candidates is already limited. Of these, not all will accept functional neurosurgery. In addition, the emerging nature of DBS as a therapy for psychiatric indications means that in many jurisdictions, government funding for device implantation and maintenance is not available, with the result that new data can only be collected through independently-funded trials.

Extending prior clinical findings, we also characterise a subcortical structural connectivity profile associated with optimal response to DBS at this target. Here, a right-hemispheric tract traversing the stimulation field and associated with YBOCS reduction connected the BNST to the amygdala. Connected fibres also involved the hippocampal formation and fornix, which form part of the circuit of Papez^45^. From a physiological perspective, the BNST functions as a component of the ‘extended amygdala’ and drives a state of sustained apprehension (anxiety), with these connectivity findings strongly suggesting that the extended amygdala is being modulated in those participants responding to BNST area DBS^46^. Of note, recent work has also demonstrated a central role for the amygdala in mediating a rapid reduction in anxiety symptoms after ALIC DBS for OCD^47^, which heralds later improvement in obsessions and compulsions. Overall, this supports the role of DBS in facilitating fear extinction through reducing anxiety. Aberrant fear conditioning (enhanced acquisition and impaired extinction) is a central construct in the development and maintenance of OCD^48,49^ and may explain why more severely affected individuals cannot tolerate or do not respond to exposure-oriented CBT^50^. This may also explain why, after DBS, our participants were now able to tolerate, and accrue a statistically-significant additional benefit from CBT during open stimulation, consistent with the previous work^31^.

Importantly, this improvement in fear extinction may be mediated via enhanced top-down input to the amygdala from the prefrontal cortex^47^. In our cohort, fibres associated with YBOCS reduction were also characterised passing to the prefrontal cortex and potentially representing a structural correlate of this effect. This connectivity profile was similar in distribution to that previously described by other centres employing different targets such as the NAcc / ALIC interface and the STN^27,51^. These findings support the existence of a common anatomical substrate that underpins response across discrete sites, as well as being consistent with prior work demonstrating that alterations in frontostriatal connectivity are implicated in response to NAcc/ALIC DBS^52^. Moreover, the distribution of connected fibres associated with YBOCS reduction was strikingly similar to prior research characterising the structural connectivity of the BNST in healthy participants^53^.

We did not find a statistically-significant association between recruitment of fibres in a pooled white matter tract atlas^29^ and reduction of OCD symptoms in our cohort. There are several potential reasons for this finding. First, our sample size was considerably smaller than the *n* = 50 participants used to develop this atlas (although it is noted the atlas has been independently validated in cohorts of similar size to our study)^44^. Second, our target in the BNST area is distinct from other surgical targets in cohorts that have contributed to the atlas and employed in validation studies (i.e. STN, ALIC and ventral striatum). As can be demonstrated in an elegant and novel study employing dual stimulation sites (STN and ALIC) in a crossover manner^28^, there are commonalities but also differences between the pattern of brain network stimulation associated with relief of OCD symptoms at each surgical site. Our finding associating amygdala and circuit of Papez connectivity with clinical response may be a connectivity profile more likely to be associated with BNST area stimulation. An important open question for the field is whether recruitment of fibres in a common ‘unified’ tract can be reliably achieved via axonal stimulation of fibres of passage, or whether it is preferable to target the axon terminals of projection fibres in a subcortical nucleus such as the STN, NAcc or STN. Recent modelling work suggests that terminating axons have lower activation thresholds than fibres of passage and are hence more excitable in response to an applied stimulus^54^. This suggests that optimal targeting of stimulation should still incorporate subcortical ‘hubs’, whilst integrating emerging connectomic findings.

Connectivity of the stimulation field with right-hemispheric cortical regions of interest in the prefrontal, temporal, parietal and occipital lobes was also significantly associated with YBOCS reduction. Interestingly, these same regions have previously been implicated in morphometric analyses of structural connectivity, grey matter volume, cortical thickness, surface area and gyrification amongst individuals with OCD^55–58^, suggesting that there may be a neuroanatomic ‘fingerprint’ of susceptibility to OCD that is modulated by DBS. Importantly, using cross-validation, YBOCS reduction could be accurately predicted in a single participant by comparing their connectivity profile to a pooled analysis of the connectivity amongst the remainder of the cohort. This suggests that the recruitment of specific fibre pathways by the stimulation field is an important determinant of outcome. More generally, the right lateralisation of our findings is interesting given previous work that implicates right-hemispheric corticostriatal circuits in inhibition^59–61^, impulsivity after subthalamic DBS for Parkinson’s disease^62,63^ and reduction of OCD symptoms after NAcc/ALIC DBS^27^. It seems unlikely that small differences in the location of active contacts between left and right hemispheres (Fig. 2C–F) was responsible for this lateralisation, given the relatively large stimulation amplitudes that afforded similar distributions of charge within both hemispheres and subcortical structures (Fig. 2B and Supplementary Figs. 2 and 3), although recognising that there are certain assumptions and simplifications inherent in the simulation of stimulation volumes. To test this finding, consideration could be given to reducing the amplitude of left hemispheric stimulation in participants responding to DBS.

Serious adverse events were predominantly accounted for by persisting psychiatric symptoms in a non-responder with repeated readmissions to hospital. It is noteworthy that the connectivity profile of this individual was most distinct from the rest of the cohort with electrodes that were more anteromedial (this was not intentional) and a stimulation field that was less connected to the right-hemispheric regions of interest (Figs. 2 and 4, Supplementary Fig. 2), suggesting a potential explanation for this lack of response. IPG infection and device removal was the most significant device-related event, affecting one participant. No participants developed major stimulation-related psychiatric side effects such as severe agitation, impulsivity and hypomania, as has previously been reported^15,17,28^. Only one participant developed mild internal agitation at higher stimulation amplitudes. This may have been attributable to our deliberately slow titration protocol and the use of lower stimulation amplitudes than have previously been described in this region. However, despite the use of more modest amplitudes, IPG depletion occurred in all participants before the close of the trial necessitating replacement.

There are a number of limitations inherent to our study design. Firstly, we did not incorporate an assessment of anxiety, focussing on the YBOCS and MADRS in this analysis. This would have been of great interest given our identified connectivity profile and will be important to evaluate in future cohorts.

The use of a staggered-onset rather than a crossover design in the double-blind phase could be considered a limitation. In previous trials using a crossover design^16–18^, optimal stimulation settings were already determined after an open-phase, increasing the likelihood of a true treatment effect in the active condition. However, based on prior work describing a significant rebound of aversive OCD symptoms after therapy interruption^64^, we considered it more ethically acceptable to delay treatment rather than cease a treatment that had previously been effective. Moreover, one significant benefit of our relatively slow approach to titrating stimulation was that the likelihood of participants becoming unblinded by sensations associated with active stimulation was minimised. On the other hand, the use of this fixed titration protocol in the blinded phase meant that effective stimulation amplitudes may not have been reached until late in the treatment period, leading to an underestimation of effect. Furthermore, the use of higher stimulation amplitudes above 4.5 Volts may have been associated with better outcomes. Ultimately, we decided that our protocol struck a balance between minimising side effects, maintaining the blind and reducing the influence of other variables outside the primary factor of interest (on versus off stimulation).

The use of normative rather than participant-specific connectivity data is a further limitation and has been discussed elsewhere^51,65,66^. Whilst participant-specific anatomical variability is lost, the quality of these group-average datasets is high and curated by teams with longstanding expertise. The reliability of analyses derived from these data may therefore be acceptable and normative connectomic data has been employed to make out-of-sample predictions across disorders and treatment modalities^21,27,67–69^. Thus, whilst normative data should not be the basis for surgical decision-making in one individual, it may yield important insights into mechanisms of disease and treatment-response within and across cohorts.

A final comment is on nomenclature. Although we refer to the BNST as our surgical target and our electrodes are distributed in a relatively tight cluster, our stimulation fields are large and encompass a relatively wide region of the subcortex. Moreover, our ventral contacts are in the superolateral hypothalamus. Additionally, the BNST is a relatively diffuse and complex structure with four subdivisions and projections to the amygdala, hypothalamus and prefrontal cortex (reviewed and illustrated in Lebow and Chen^46^). However, the parallels with our connectivity findings and the known connectivity profile of the BNST is striking and indicates that modulation of this structure is indeed associated with the response of our participants. Nevertheless, this and recent overlapping work characterising fibre tracts associated with symptom reduction^27–29^ suggests that it may be more appropriate to refer to and target fibre bundles rather than isolated anatomical regions. This is consistent with the idea of using neuromodulation to target distributed brain networks and can be advanced with the increasing precision afforded by normative and patient-specific connectomic data^70^. Hence we refer to the BNST region in this manuscript, as we cannot be conclusively certain that the BNST is the only subcortical structure of relevance to our clinical findings and it may be the fibre connectivity profile of active stimulation that is the mediator of clinical benefit.

In summary, in a cohort of participants with severe, treatment-refractory OCD, we demonstrate that active stimulation at the BNST region is superior to placebo in a randomised, double-blind, sham-controlled, delayed-onset clinical trial, with a further significant benefit accrued following a longer phase of open-label stimulation incorporating a course of ERP-based CBT. We also delineate a structural connectivity profile associated with clinical response, which comprised subcortical regions implicated in fear conditioning and emotional processing, as well as cortical regions implicated in prior morphometric analyses of persons with OCD. We anticipate that our findings will motivate more precise targeting of stimulation within these networks, using participant-specific connectivity data to optimise treatment at the individual level, as has been described in DBS for treatment-resistant major depression^71,72^.

## Supplementary information

Supplementary Material
